# Photosensitizer Micelles Together with IDO Inhibitor Enhance Cancer Photothermal Therapy and Immunotherapy

**DOI:** 10.1002/advs.201700891

**Published:** 2018-02-26

**Authors:** Jinrong Peng, Yao Xiao, Wenting Li, Qian Yang, Liwei Tan, Yanpeng Jia, Ying Qu, Zhiyong Qian

**Affiliations:** ^1^ State Key Laboratory of Biotherapy and Cancer Center West China Hospital Sichuan University, and Collaborative Innovation Center Chengdu 610041 Sichuan P. R. China; ^2^ Department of Pharmacy West China Second University Hospital No. 20, People's Southern Road Chengdu 610041 Sichuan P. R. China; ^3^ School of Pharmacy Chengdu Medical College No. 783, Xindu Avenue, Xindu District Chengdu 610500 Sichuan P. R. China

**Keywords:** combinational therapy, indoleamine 2,3‐dioxygenase (IDO) inhibitors, immunotherapy, ineffective photothermal therapy, nanoparticles

## Abstract

The therapeutic outcome of photothermal therapy (PTT) remains impeded by the transparent depth of light. Combining PTT with immunotherapy provides strategies to solve this problem. Regulating metabolism‐related enzymes is a promising strategy to stimulate immune response. Here, a nanosystem (NLG919/IR780 micelles) with the properties of photothermal conversion and regulation of the tryptophan metabolic pathway is used to suppress the growth of the tumor margin beyond effective PTT and promote tumor PTT and immunotherapy. It is revealed that mild heat treatment promotes the growth of the tumor margin beyond effective PTT for the upregulation of heat shock protein (HSP), indoleamine 2,3‐dioxygenase (IDO), and programmed death‐ligand 1 (PD‐L1). The NLG919/IR780 micelles can effectively inhibit the activity of IDO but do not affect the level of IDO expression. NLG919/IR780 micelles can effectively accumulate in the tumor and can migrate to lymph nodes and the lymphatic system. In vivo antitumor studies reveal that NLG919/IR780 micelles effectively suppress the growth of tumor margin following PTT in primary tumors. NLG919/IR780 micelle‐mediated PTT and IDO inhibition further stimulate the activation of T lymphocytes, inhibiting the growth of distal tumors (abscopal effect). The results demonstrate that the NLG919/IR780 micelles combine PTT and immunotherapy and suppress the tumor margin as well as distal tumor growth post photothermal therapy.

## 1 Introduction

The emergence of nanomedicines provides an alternative strategy for the delivery of therapeutic agents.^1^ The ongoing development of nanoparticles not only improves the targeting and bioavailability of drugs and develops new therapeutic methods, such as photothermal therapy and nanovaccines, but also provides a suitable carrier for the realization of combination therapy.^2^, ^3^, ^4^, ^5^, ^6^, ^7^ Moreover, some nanoparticle‐based systems can strengthen the immune response in tumor immunotherapy, which has attracted much attention.^8^, ^9^, ^10^, ^11^

During cancer therapy, some therapeutic approaches such as chemotherapy and radiotherapy inhibit the primary tumor growth to a certain extent. Additionally, apoptosis or necrosis of cancer cells induced by these therapies can produce enormous amounts of cell debris, which contains neoantigens sufficient to stimulate the immune system of the host and generate an immune response, thereby inhibiting the growth of distal tumors.^12^ This phenomenon is termed an “abscopal effect.” However, the abscopal effect induced by approaches such as chemotherapy, radiotherapy, or hyperthermia is weak and does not last; therefore, the growth inhibition of distal tumors mediated by the abscopal effect needs to be further strengthened.^13^ Conventional nanomedicines have improved the drug delivery efficiency and reduced side effects but have no effect on enhancing the abscopal effect.^14^ Multifunctional nanoparticles and combination therapies are required to enhance this effect.

Studies have shown that during radiotherapy, additional administration of nanoparticles that can specifically absorb neoantigens can enhance the efficacy of antigen‐presenting cells (APCs) in antigen presentation, which further strengthens the abscopal effect and increases the growth inhibition of distal tumors.^15^ Similar to photodynamic therapy, which can enhance the outcome of checkpoint blockage therapy, a combination of photothermal therapy (PTT, another photo‐induced therapeutic manner) with immune stimulation mediated by carbon nanotubes can enhance the immune response with the subsequent administration of anti‐CTLA‐4.^16^, ^17^, ^18^, ^19^ Moreover, the introduction of an immunomodulator (R837), combined with PTT and an immunological checkpoint inhibitor, can not only enhance the abscopal effect but can also enhance the immune memory of the host, providing the host with long‐term immune recognition to tumor cells.^20^ These results not only demonstrate that the PTT is an efficient therapeutic approach for inhibiting primary tumors but also illustrate its potential to inhibit distal tumor growth when combined with other therapies.^21^, ^22^, ^23^ Therefore, the combination of multifunctional nanoparticles with multiple therapeutic approaches is a promising means to achieve hypothetical antitumor response in tumor immunotherapy.

Although nanoparticle‐mediated PTT has been highlighted in recent years for its efficacy and immune system stimulation, the therapeutic outcome remains limited by the penetration depth of light (several millimeters even with near infrared (NIR) laser).^24^ While the tumor size is larger than the laser penetration limitation, the tumor margin beyond effective PTT leads to further growth of the tumor. The PTT‐induced “abscopal effect” is still too weak to suppress the growth of the remaining tumor margin. The combination of PTT with immunomodulation has been reported to demonstrate potential in cancer therapy.^16^, ^17^, ^18^, ^19^, ^20^ Furthermore, the tumor margin beyond effective PTT undergoes mild heating. Mild heating can upregulate some proteins for cell protection, such as heat shock protein (HSP); HSP inhibitors can enhance the therapeutic efficacy of PTT.^25^ In addition, heating regulates the expression of not only HSP but also other proteins. Inhibition of some of these proteins that strengthen the immune escape of tumor cells can be an effective strategy to suppress the growth of the tumor margin beyond effective PTT and secondary (distal) tumors. Among these proteins, those altering the metabolic behavior of tumor cells or immune cells provide an alternative choice.

Regulating the immune response by altering the metabolism of immune cells is currently an attractive strategy.^26^ Modifying the IDO (indoleamine 2,3‐dioxygenase) functions in tryptophan metabolism is one strategy. Studies have shown that IDO is an immunomodulator that inhibits T lymphocyte activity and produces immune tolerance to tumor cells.^27^ Several studies indicate that IDO aids cancer cells in escaping from the immune system during tumor growth and metastasis.^28^, ^29^, ^30^ Additionally, it has been proven that high expression of IDO in APCs inhibits APC activity and reduces antigen‐presenting efficacy.^31^ Therefore, regulation of IDO functions has great significance in regulating immune response to tumors. To date, the effect of heat treatment on the expression of IDO in tumor cells has not been evaluated, and the effect of IDO inhibition on suppression of the tumor margin beyond PTT and cancer immunotherapy is unclear.

**Scheme 1:**
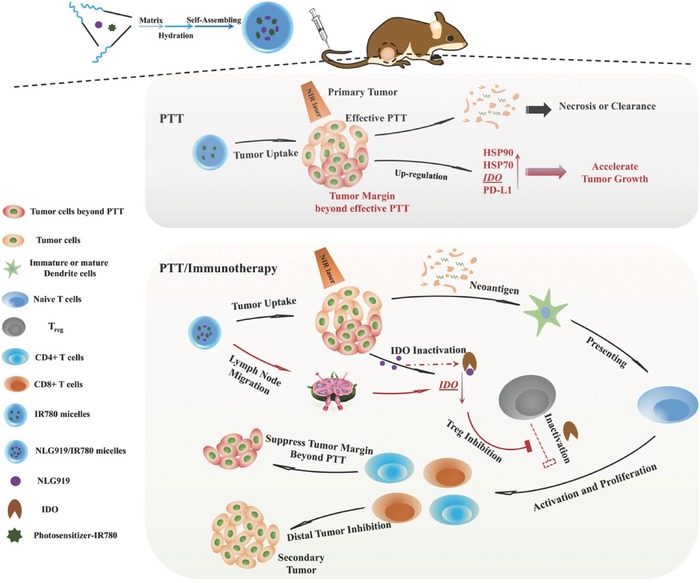
Scheme of the preparation pathway of NLG919(IODI)/IR780 coloaded micelles and the mechanism by which the NLG919/IR780‐micelle‐mediated PTT combined with immunotherapy suppressed the growth of the tumor margin beyond effective PTT and the distal (or secondary) tumor. When the tumor was treated with PTT, only several‐millimeter‐deep tumor tissue undergoes effective PTT. The remaining tumor tissue (tumor margin) undergoes an ineffective PTT, in which HSP, IDO, and PD‐L1 are upregulated, accelerating the growth of the tumor margin. Introduction of an IDO inhibitor can combine with the PTT, resulting in IDO‐inhibition‐mediated immunomodulation to further suppress the growth of the tumor margin beyond effective PTT, as well as distal tumor.

In our previous work, we observed that PTT can efficiently inhibit the growth of primary tumors^32^, ^33^, ^34^, ^35^ and further confirmed that photothermal therapy can effectively enhance chemotherapy.^36^, ^37^ The enhancement may be chiefly related to the immune response induced by PTT. It is expected that the additional introduction of an IDO inhibitor can further enhance the immune response induced by PTT. However, photothermal therapy combined with IDO inhibitors has not been evaluated in the growth of tumors, particularly distal tumors. To our knowledge, most IDO inhibitors (NLG919, or Epacadostat, etc.) are hydrophobic, which limits their bioavailability and biofunctions. Their prodrugs have been reported recently, but micellar formulations of these IDO inhibitors have not yet been reported.^38^ We have used the amphipathic polymer MPEG‐PCL to effectively load chemotherapeutic drugs, immunosuppressive agents and near‐infrared dyes and have developed a series of nanomedicines.^39^, ^40^, ^41^ In this study, based on our previous studies, we plan to first evaluate the effect of heat treatment on the protein expression and growth of tumor cells in vitro and in vivo and then construct a nanosystem (NLG919/IR780 micelles) possessing the properties of photothermal conversion and tryptophan metabolic pathway regulation. We aim to investigate the effect of this nanosystem on suppressing the growth of the tumor margin beyond PPT and enhancing tumor immunity (**Scheme** **1**). IR780 and NLG919 were selected as the photosensitizer and IDO inhibitors, respectively. Because APCs are primarily localized at the lymphatic systems, including lymphatic nodes, the nanoparticles need to enter the lymphatic system to inhibit the IDO activity. Therefore, we further evaluated the properties of the migration of NLG919/IR780 to the tumor and lymphatic nodes. Finally, the effect of the NLG919/IR780 micelles on IDO function, the tumor growth inhibition mediated by PTT and immunotherapy and the mechanism of immune therapy were investigated in detail.

## 2 Results and Discussion

### 2.1 Heat Treatment Accelerated Growth of the Tumor Margin Beyond PTT

**Figure 1:**
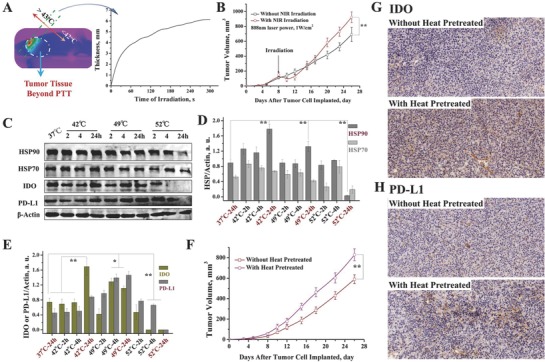
Heat treatment accelerated the growth of the tumor margin beyond PTT. A) The depth of effective PTT. Above 43 °C, tumor cell can be effectively suppressed. B) Tumor volume of the mice treated with saline and with PTT (partially treated). C) Protein characterization of 4T1 cells with different heat treatments (treated with heat for 5 min then cultured with different times). D) Proportion of variation of HSP90 and HSP70 after different heat treatments. E) Proportion variation of IDO and PD‐L1 after different heat treatments. F) Tumor volume of the mice implanted with normal 4T1 cells and preheated 4T1 cells (42 °C for 5 min, then cultured for another 24 h before implantation). G,H) The images of IHC analysis performed on representative subsets of the tumors (G, IDO; H, PD‐L1, tumor tissues were obtained after the experiment in Figure 1F was completed). Magnification: 20×. The results of ICH analysis further proved the upregulation of IDO and PD‐L1 in the tumor tissues in which the tumor cells were preheated before the implantation.

Due to the penetration limitation of NIR light and heat diffusion, when the tumor size is larger than this limitation, part of the tumor margin beyond effective PTT undergoes mild heating (below 42 °C). In our previous report, we have revealed that upon irradiation with 808 nm laser, the thickness of the tumor tissue portion heated to greater than 43 °C is smaller than 6 mm, even when the highest temperature is over 72 °C (**Figure** **1**A). We further compared the growth rates of the tumor margin beyond effective PTT and that for the tumor, following PTT. The results show that the tumor margin beyond effective PTT was proliferating faster than tumors not subjected to PTT (Figure 1B). It indicates that ineffectiveness of the PTT accelerated the tumor growth. Based on these phenomena, the effect of heat treatment on the protein expression of tumor cells was evaluated. In addition to HSP (HSP90, HSP70), IDO and programmed death‐ligand 1 (PD‐L1) were upregulated after heat treatments (42 °C or 49 °C, 5 min) (Figure 1C–E). When the heat treatment reached a temperature of 52 °C, HSP, IDO, and PD‐L1 were all significantly downregulated (Figure 1C–E). The downregulation of these proteins may be ascribed to heat‐induced degradation while the temperature was sufficiently high. We further evaluated the duration of expression of the upregulated proteins, such as IDO, PD‐L1, and HSP. We found that with time, the expression of IDO increased at 48 h after the heat treatment and then decreased at 72 h. However, the expression of PD‐L1 increased with time (highest expression at 72 h) (Figure S1, Supporting Information). It has been proven that the upregulation of HSP, IDO, and PD‐L1 is associated with immune escape and tumor metastasis. It indicates that ineffectiveness of PTT may accelerate tumor growth. To prove this, we implanted the 4T1 tumor cells, pretreated in a 42 °C environment, into balb/C mice. We compared the tumor growth rate of the preheated tumor cells with that of tumor cells that were not preheated. The results revealed that the preheated 4T1 cells were proliferating faster than the cells that were not preheated (Figure 1F, Supporting Information). From these results, we can conclude that mild heating can accelerate the growth rate of tumor cells. The upregulation of HSP, IDO, and PD‐L1 is an additional reason for the faster proliferation of the tumor margin beyond effective PTT, compared with the untreated tumors. Inhibiting the activity of these proteins may favor suppression of growth of the tumor margin beyond effective PTT.

### 2.2 Preparation and Characterization of NLG919/IR780 Micelles

**Figure 2:**
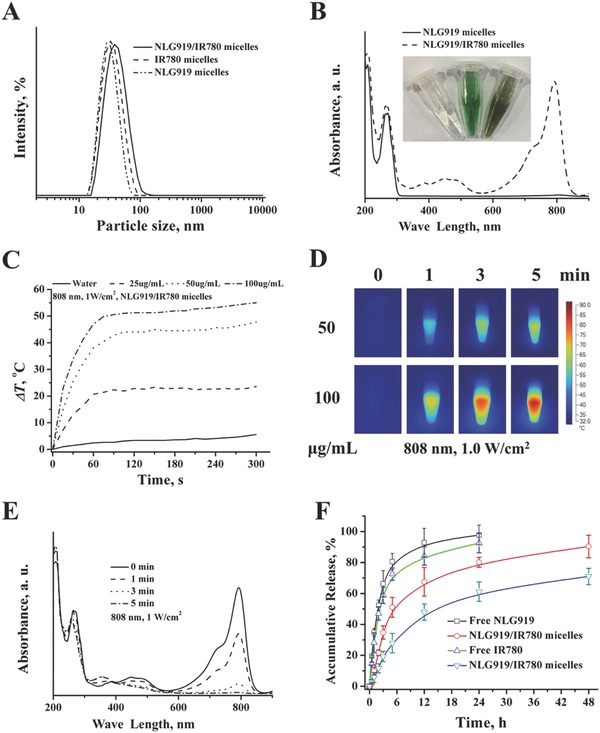
Characterization of NLG919/IR780 micelles. A) Particle size distribution of NLG919/IR780 micelles, NLG919 micelles, and IR780 micelles. B) UV–vis spectrums of NLG919/IR780 micelles, NLG919 micelles. C) Photothermal conversion of NLG919/IR780 micelles in vitro. D) IR thermal images of NLG919/IR780 micelles dispersion. E) UV–vis spectrums of NLG919/IR780 micelles under the irradiation of 808 nm laser. F) Release behavior of NLG919 and IR780 from NLG919/IR780 micelles, respectively.

*Structure Characterization of NLG919/IR780 Micelles*: Some studies have been performed to inhibit the activity of HSP and enhance the therapeutic outcome of PTT. However, most HSP inhibitors cannot stimulate the host to generate the required immune response for cancer immunotherapy. Therefore, we chose an IDO inhibitor and aimed to construct IDO inhibitor/photosensitizer coloaded micelles to overcome the low bioavailability caused by their inherent hydrophobicity and develop an efficient combination therapy. NLG919/IR780 micelles were prepared by the thin‐film rehydration method, as reported in our previous studies (Scheme 1). The particle size and distribution were measured by dynamic light scattering (DLS). The particle size of NLG919/IR780 was 43 ± 3.2 nm, which is slightly larger than NLG919 micelles (30 ± 2.4 nm) and IR780 micelles (29.6 ± 2.8 nm) (**Figure** **2**A). NLG919/IR780 micelles are smaller than 50 nm, which is proved to be suitable for lymphatic circulation.^42^ We further investigated the optical properties of NLG919/IR780 micelles. Green or dark blue‐green appearance indicated the absorption of IR780 micelles or NLG919/IR780 micelles in the NIR region. The UV–vis spectrum of NLG919/IR780 micelles further proved the presence of NLG919 (265 nm) and IR780 (793 nm) (Figure 2B).

*Photothermal Conversion*: Due to the strong absorption of IR780 in the NIR region, it can be used as a medium for photothermal conversion. We first investigated the photothermal conversion of the NLG919/IR780 micelles in PBS (pH = 7.4). When the concentration of the micelles was 25 µg mL^−1^, the temperature of the aqueous dispersion rose from 25 to 44.9 °C in 1 min under irradiation with a 808 nm laser. As the concentration increased to 50 and 100 µg mL^−1^, the temperature reached 63.7 and 77 °C, respectively (Figure 2C). The results indicate that the NLG919/IR780 micelles can be used as a suitable medium for PTT. The IR thermal imaging visualized the process of heat generation, which further proved the photothermal conversion ability of the NLG919/IR780 micelles (Figure 2D). Following the laser irradiation, the absorption of the NLG919/IR780 micelles in the NIR region decreased dramatically. With prolonged irradiation, the absorption decreased further (Figure 2E), indicating photo bleaching. Photo‐bleaching may cause drawbacks in the photothermal performance of the NLG919/IR780 micelles; however, the temperature of the NLG919/IR780 micelle dispersion can be maintained at a steady value, and the equilibrium values can be controlled by adjusting the concentration of the micelles. Thus, photobleaching may prevent overheating during PTT.

*Drug Release Behaviors*: The release behavior of NLG919 and IR780 was evaluated. Compared with the free form of NLG919 or IR780, the release rates of NLG919 or IR780 from the NLG919/IR780 micelles were slow (Figure 2F). This indicates that the NLG919/IR780 micelles exhibited delayed drug release behavior. The slow release can enhance the efficiency of drug delivery of the NLG919/IR780 micelles to the target tissues, such as tumor sites or the lymphatic circulation.

### 2.3 IDO Activity Inhibition and Photothermal‐Mediated Growth Inhibition of Tumor Cells In Vitro

**Figure 3:**
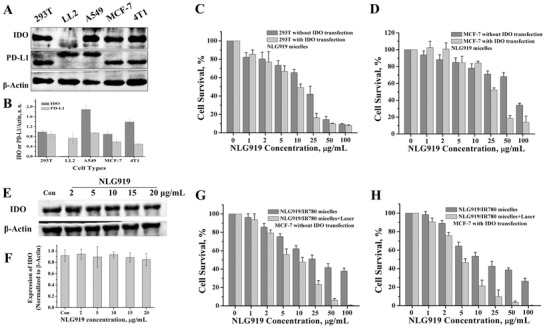
IDO activity inhibition mediated by NLG919 micelles and growth inhibition of tumor cells cotreated with IDO inhibition and PTT. A,B) IDO and PD‐L1 expression of different cell types. C) 293T cell survival after coculturing with NLG919 micelles with or without IDO gene transfection. D) MCF‐7 cell survival after coculturing with NLG919 micelles with or without IDO gene transfection. E,F) Effect of NLG919 micelles on the expression of IDO after treatment. G) MCF‐7 cell survival after coculturing with NLG919/IR780 micelles with or without laser irradiation (without IDO transfection). H) MCF‐7 cell survival after coculturing with NLG919/IR780 micelles with or without laser irradiation (with IDO transfection).

Multiple types of normal cells (DC, macrophage, etc.) and tumor cells overexpress IDO. Before evaluating IDO inhibition by NLG919 micelles, we first identified the expression level of IDO in different cell types. 293T, LL2, A549, MCF‐7, and 4T1 cell lines were chosen. The results revealed that 293T, A549, MCF‐7, and 4T1 showed high expression of IDO, and IDO was detected in LL2 cells (**Figure** **3**A,B). Next, we chose 293T and MCF‐7 cell lines to evaluate the inhibition of IDO activity in vitro and chose 4T1 cells to establish a cancer model in vivo. We first investigated whether the NLG919 micelles could suppress the activity of IDO in the 293T and MCF‐7 cell lines. The IC_50_ of the NLG919 micelles in the 293T and MCF‐7 cells were 20 and 75 µg mL^−1^ (NLG919 concentration), respectively (Figure 3C,D). The cell lines were transfected with the IDO gene via liposome 3000 to obtain 293T and MCF‐7 cell lines overexpressing IDO. The IC_50_ values of the NLG919 micelles in the IDO‐transfected 293T and MCF‐7 cell lines were 11 and 32 µg mL^−1^, respectively (Figure 3C,D). The results indicate that the NLG919 micelles can efficiently inhibit the activity of IDO. However, when we further measured IDO expression in the NLG919‐micelle‐treated MCF‐7 cells, no obvious downregulation of IDO was observed (Figure 3E,F). This indicates the NLG919 micelles inhibit not the expression of IDO but the activity of IDO, which lowers the cell survival of cell lines expressing high IDO levels.

We then evaluated the inhibition of tumor cell growth by NLG919/IR780‐micelle‐mediated PTT. Without IDO transfection, the IC_50_ values of the NLG919/IR780 micelles and the NLG919/IR780 micelles + laser were 26 and 7.8 µg mL^−1^, respectively. Following IDO transfection, the IC_50_ values of the NLG919/IR780 micelles and the NLG919/IR780 micelles + laser were 14.3 and 4.3 µg mL^−1^, respectively (Figure 3G,H). The results illustrate that NLG919/IR780‐micelle‐mediated PTT can inhibit the proliferation of MCF‐7 efficiently. Previous reports have demonstrated that the upregulation of HSP70, HSP90, PD‐L1, or IDO suppresses the activation of cytotoxic T cells. Therefore, photothermal therapy alone may be unable to efficiently suppress the growth of the tumor margin beyond effective PTT and distal tumors. Thus, it is reasonable to introduce an IDO inhibitor to invigorate the activity of effector T cells.

### 2.4 Tumor Targeting and Suppression of the Tumor Margin Beyond Effective PTT of NLG919/IR780 Micelles

**Figure 4:**
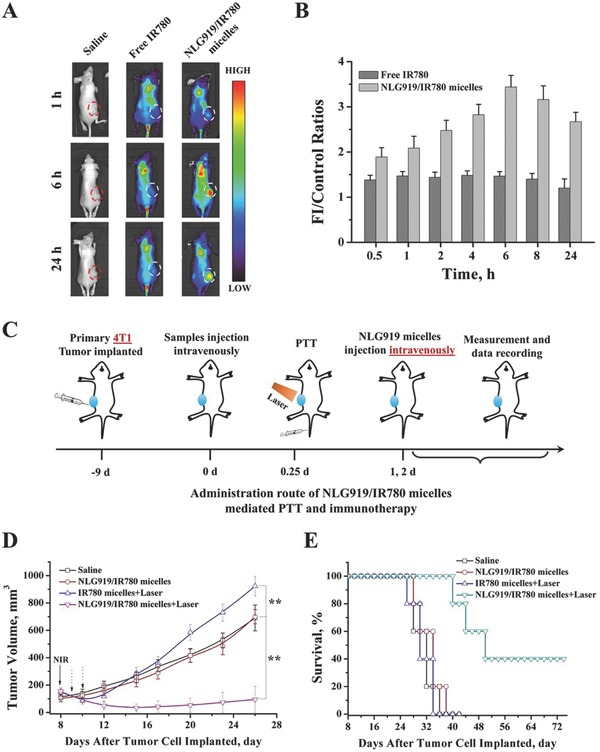
Passive tumor targeting of NLG919/IR780 micelles and suppression of the growth of the tumor margin beyond PTT. A) Fluorescence images of MCF‐7 breast cancer‐bearing balb/C‐nu mice treated with saline, free IR780, and NLG919/IR780 micelles. B) The fluorescence intensity of tumors versus time. Saline, free IR780, and NLG919/IR780 micelles were injected intravenously. C) The administration route of NLG919/IR780 micelles to evaluate the growth inhibition mediated by PTT and IDO inhibition to the tumor margin beyond PTT. D) Tumor volume of the mice with different treatments. E) Mean survival of the mice with different treatments.

*Tumor Targeting*: The tumor targeting ability of NLG919/IR780 micelles decides the outcome of PTT‐mediated tumor growth inhibition in the primary tumors and the consequent immune modulation by inhibition of IDO activity that suppresses the growth of the tumor margin beyond effective PTT. We first studied the tumor targeting of the NLG919/IR780 micelles following intravenous injection. The fluorescence intensity of the tumor site in the NLG919/IR780‐micelle group was much stronger than that in the free‐IR780‐micelles group. The fluorescent intensity (FI) for the former group reached a maximum value at the 6th time point, which was approximately twice as that in the free‐IR780‐micelle‐treated group (**Figure** **4**A,B). This indicates that the NLG919/IR780 micelles have passive targeting properties via the EPR effect, resulting in the high accumulation of the photosensitizer and NLG919 in the tumor site. Furthermore, we evaluated the pharmacokinetics of the NLG919/IR780 micelles by measuring the concentration variation of NLG919 in the blood via LC‐MS (Figure S2, Supporting Information). The pharmacokinetic parameters are listed in **Table** **1**. Apparently, compared with the free form of NLG919, the NLG919 micelles improved the AUC and t_1/2_ of NLG919, which could enhance the therapeutic outcome of NLG919.

**Table 1:** Pharmacokinetic parameters of NLG919 in different formulations

Groups	t_1/2_ [h]	AUC [µg h mL^−1^]	C_max_ [µg mL^−1^]	CL [mL h^−1^ kg^−1^]
NLG919/IR780 micelles	3.28	37.10	8.49	5.32
Free NLG919	1.03	5.90	8.13	44.24

*Suppression of Tumor Margin Beyond Effective PTT*: We next established a 4T1 breast cancer model in balb/c mice to evaluate the suppression of the tumor margin beyond effective PTT. When the tumor volumes of the mice reached ≈240 mm^3^ (≈8 mm × ≈8 mm), the mice were divided and administered different treatments (Figure 4C). Following the combination of PTT with the IDO inhibitor micelle treatment, regression was observed in the tumor margin beyond effective PTT (Figure 4D). The survival of the mice treated with PTT plus IDO inhibitor micelles was greatly prolonged (Figure 4E). This indicates that the introduction of IDO inhibitor during PTT can suppress the growth of the tumor margin beyond effective PTT and enhance the therapeutic outcome of PTT.

### 2.5 PTT/IDO‐Activity Inhibition Suppresses the Primary Tumor and Secondary Distal Tumors In Vivo

**Figure 5:**
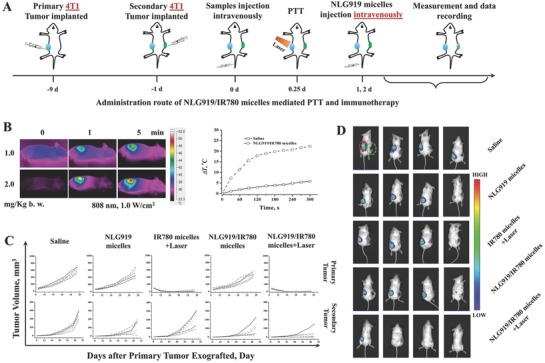
4T1 Tumor cell growth inhibition mediated by NLG919/IR780‐micelle‐based PTT and immunotherapy. A) Administration route of NLG919/IR780‐micelle‐mediated PTT and immunotherapy. B) Photothermal conversion of NLG919/IR780 micelles in vivo. Right) Infrared thermal images of mice treated with 1.0 and 2.0 mg kg^−1^ b.w. Left) Surface temperature of tumor site versus irradiation time (IR780: 2.0 mg kg^−1^ b.w.). C) Tumor volume of primary tumor and secondary tumor versus time, respectively (*n* = 6). D) Images of the tumor‐bearing mice 26 d after the primary tumor cells were implanted. (Right: primary tumor, green circle, 4T1 tumor. Left: secondary tumor, red circle, 4T1‐LUC tumor.)

*Tumor Growth Inhibition*: The NLG919/IR780 micelles showed effective inhibition of IDO activity, which could improve the inhibition of tumor cell growth in vitro. They additionally exhibited the desired passive tumor targeting and suppressed the tumor margin beyond effective PTT in vivo. As IDO inhibitors are also used as immunomodulators, the effect of IDO activity inhibition combined with PTT on cancer immunotherapy needs to be further investigated. Therefore, a 4T1 breast cancer model was established on the balb/c mice by a direct subcutaneous injection of 4T1 cells to the right flanks of the mice. Eight days later, the primary tumor volumes reached 100 mm^3^. Next, we subcutaneously injected 4T1 cells to the left flank of the mice and investigated the effect of the treatments on the tumor growth in vivo. The administration route is illustrated in **Figure** **5**A. After the second injection of 4T1 cells, the mice were divided into five groups and treated with saline, the NLG919 micelles, the IR780 micelles + laser, the NLG919/IR780 micelles, or the NLG919/IR790 micelles + laser. The photothermal conversion of the NLG919/IR780 micelles was evaluated by infrared thermal imaging. After administration of 1 mg kg^−1^ b.w. of the NLG919/IR780 micelles, under irradiation with the 808 nm laser, the surface temperature of the tumor reached 45 °C. When the dosage was increased to 2 mg kg^−1^ body weight (b.w.), the surface temperature further increased to 54 °C. The surface temperature of the mice treated with saline only increased to 41 °C (Figure 5B). This demonstrates that the NLG919/IR780 micelles can be used to mediate PTT. In the case of primary tumor treatments, the administration of NLG919 inhibited tumor growth, but not significantly. With the introduction of laser irradiation in the groups treated with the IR780 micelles and NLG919/IR780 micelles, tumor growth was inhibited efficiently, and some tumors were eliminated. No significant differences were found between these two groups. In the case of secondary tumors, treatment with NLG919 without laser irradiation inhibited the growth of secondary tumors more efficiently than that in the group treated with IR780 micelles + laser irradiation (Figure 5C). The group treated with NLG919 and PTT (NLG919/IR780 micelles + laser) demonstrated the best therapeutic outcome for inhibition of secondary tumor growth. We further established the 4T1‐LUC tumor model to visualize the growth of secondary tumors. The results further confirmed the therapeutic outcome of PTT/immunotherapy (Figure 5D).

**Figure 6:**
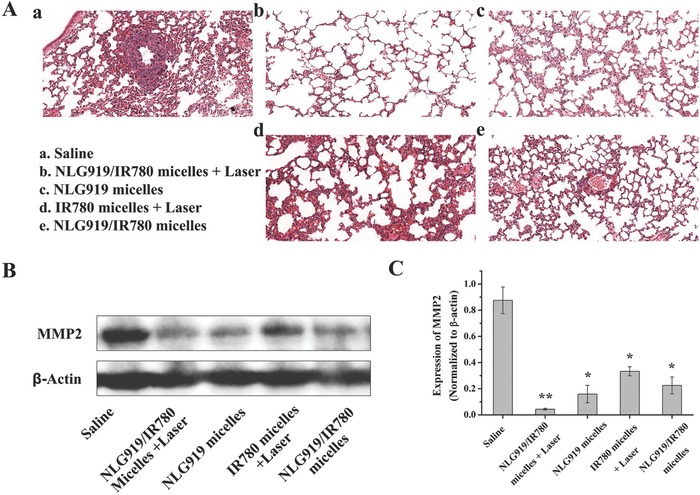
Lung metastasis alleviation. A) The HE staining subsets representative of the lung tissues after different treatments. B,C) The MMP2 expressions in the lung tissues after different treatments.

Furthermore, the combination of PTT and IDO inhibition alleviated lung metastasis from 4T1 breast cancer. Tumor metastasis was formed in the saline‐treated mice (**Figure** **6**A). Metastasis was additionally detected in the mice treated with NLG919 micelles, the IR780 micelles + laser, and the NLG919/IR780 micelles (Figure 6B,C). The expression of MMP2 with the combination therapy is much lower than the other groups, the expression levels in which are in turn lower than that in the saline‐treated groups. This indicates that the combination treatment can alleviate lung metastasis of 4T1 tumors.

**Figure 7:**
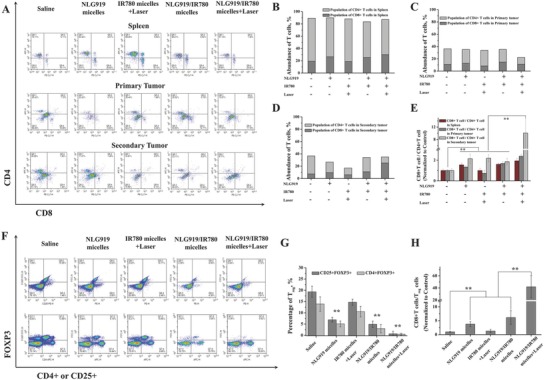
The mechanism study. Representative flow cytometry plots showing A) CD4^+^ T cells and CD8^+^ T cells in spleen, primary tumors, and secondary tumors 16 d after the treatments. B–D)The populations of CD4^+^ and CD8^+^ T cells in the tumors after different treatments. E) The ratios of CD8^+^ T cells/CD4^+^ T cells in spleen, primary tumors, and secondary tumors. Tumor cell suspensions were analyzed by flow cytometry for T‐cell infiltration (gated on CD3^+^ T cells). Populations of tumor‐infiltrating T cells (CD4^+^ T cells, CD8^+^ T cells and CD8^+^/CD4^+^) in primary tumors upon various treatments (C). F) The populations of regulatory T cells (T_reg_, CD25^+^CD4^+^FOXP3^+^) in the spleens after various treatments. G) The percentage of T_reg_ in spleens. H) The ratios of CD8^+^ T cells/T_reg_. **p < 0.01.

*Mechanism Study*: From the studies on the secondary tumor growth and metastasis inhibition, we can deduce that the therapeutic outcome may be associated with the immune response induced by the NLG919/IR780 micelles. Therefore, we further measured the proportion of infiltrated T cells in the spleen and primary and secondary tumors with different treatments. The spleens and tumor tissues were eviscerated on the 16th day after the secondary tumor cell injection. The proportions of the infiltrated T cells were identified by flow cytometry (gated on CD3^+^ T cells) (**Figure** **7**A). We further identified the populations of CD4^+^ T cells and CD8^+^ T cells in the spleen, primary and secondary tumors, respectively. In the spleen and primary tumor, the populations of CD8^+^ T cells were ≈1.8‐ and ≈2.3‐fold that of CD4^+^ T cells in the group treated with the NLG919/IR780 micelles + laser, while no significant enhancement in the population of CD8^+^ T cells was found in the other groups, with no significant difference compared with the saline‐treated group (Figure 7B–E). This indicates that the cotreatment with PTT and the IDO inhibitor can populate the CD8^+^ T cells, which could strengthen the immunotherapy for cancer. Additionally, in the secondary tumors, the population of CD8^+^ T cells was ≈10‐folds that of CD4^+^ T cells in the group treated with the NLG919/IR780 micelles + laser, and no significant enhancement in the population of CD8^+^ T cells was found in the other groups, with no significant difference compared with the saline‐treated group (Figure 7E). This indicates that the NLG919/IR780 micelles + PTT induces differentiation of T cells to CD8^+^ T cells, aiding in inhibiting the growth of the tumor. Moreover, previous reports indicate that the IDO inhibition can downregulate the function of regulatory T cells (T_reg_). We further evaluated the population of T_reg_ in the spleens after the different treatments. By direct injection of the NLG919 micelles or the NLG919/IR780 micelles, the population of T_reg_ in the spleen of the tumor‐bearing mice was decreased from ≈19% to ≈5%. No significant decrease was found in the group treated with the IR780 micelles + laser. With the combination therapy, the population of T_reg_ was further decreased to ≈0.5%. The ratios of CD8^+^ T cells/T_reg_ was increased more than 20‐fold compared with the saline‐treated groups (Figure 7F–H). Therefore, downregulation of the functions of T_reg_ may additionally aid in strengthening the tumor immune response to inhibit tumor growth.

**Figure 8:**
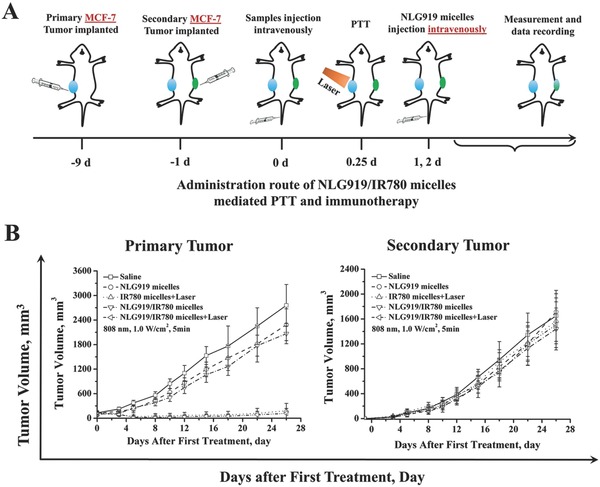
MCF‐7 tumor cell growth inhibition mediated by NLG919/IR780 micelles. A) Administration route of NLG919/IR780‐micelle‐mediated PTT and immunotherapy. B) Tumor volume of primary tumor and secondary tumor versus time, respectively (*n* = 5). The knockdown of thymus in balb/C‐nu mice leads to depletion of T cells, which limits the immunotherapeutic outcome mediated by NLG919/IR780 micelles in the secondary tumor. It also demonstrates that the activation of T cells is the main mechanism underlying NLG919/IR780‐micelle‐mediated PTT/immunotherapy.

Based on these results, we further established a MCF‐7 breast cancer model in balb/c‐nu mice to determine whether the activation of T cells is the major mechanism underlying the effect of the NLG919/IR780‐micelle‐mediated immunotherapy (**Figure** **8**A). The thymus has been knocked out in balb/c‐nu mice, which leads to the depletion of T cells. Tumor regression can still be found in the group treated with NLG919/IR780‐micelle‐mediated PTT (primary tumor). Regarding the secondary tumor, no significant difference has been found between the group treated with the NLG919/IR780 micelles + laser and the saline‐treated group, as well as in the other three groups (NLG919 micelles, IR780 micelles + laser, NLG919/IR780 micelles, respectively) (Figure 8B). This indicates that the activation of T cells mediated by NLG919/IR780 micelles + laser enables the immunotherapy of the secondary tumor, as well as PTT of the primary tumor.

### 2.6 Lymphatic Node Migration of NLG919/IR780 Micelles and Suppression of Primary Tumor and Secondary Distal Tumor In Vivo after Subcutaneous Administration

**Figure 9:**
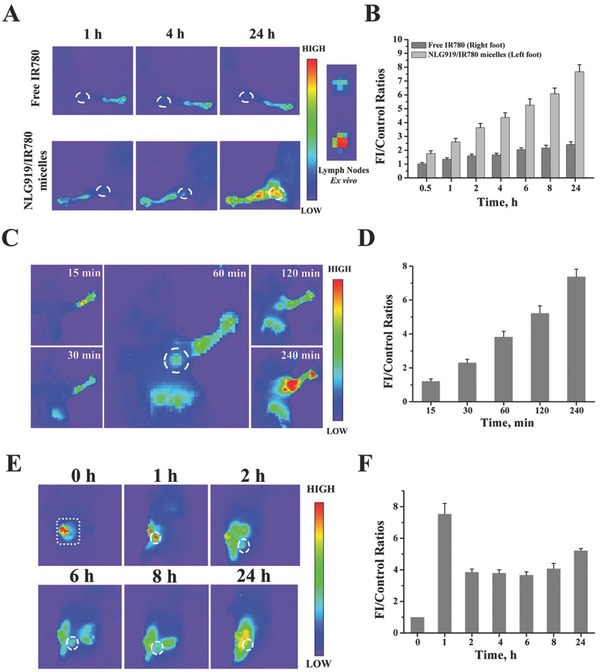
Migration of NLG919/IR780 micelles to lymphatic nodes. A) Right: migration of NLG919/IR780 micelles to *Ln. popliteus*. Left: *Ln. popliteus* tissues eviscerated at 24 h. B) The FI of *Ln. popliteus* versus time in vivo. C) Migration of NLG919/IR780 micelles to *Ln. axillaris accessorius*. D) The FI of *Ln. axillaris accessorius* versus time in vivo. E) Migration of NLG919/IR780 micelles from tumor site to *Ln. subiliacus*. F) The FI of *Ln. subiliacus* versus time in vivo. NLG919/IR780 micelles were injected intratumorally.

*Lymph Node Migration*: It has been reported that the IDO is also overexpressed in immune cells such as DCs and T cells, which are primarily localized in the lymphatic system. If we can stimulate the immune cells by subcutaneously administering the NLG919 micelles following NLG919/IR780‐micelle‐mediated PTT, it would improve the convenience of administration. Therefore, we indirectly evaluated the migration of NLG919/IR780 micelles to the lymphatic system, particularly the lymphatic nodes. The NLG919/micelles were injected into the fat patches of the feet, the hand and the tumor of the mice, respectively, and the FI of the nearest lymphatic node were detected and visualized by an IVIS spectrum in vivo imaging system (PerkinElmer). After being injected into the fat patch of the mice foot, a strong and invasive FI signal was detected in *Ln. popliteus*, which was stronger than the free‐IR780‐treated group. The quantitative FI further proved that the NLG919/IR780 micelles were migrating more easily than the free form of NLG919/IR780 in the lymphatic system and in enrichment in the lymphatic nodes (**Figure** **9**A,B). In the case of injection into the fat patch of the hands, we could visualize the unambiguous fluorescent signal in *Ln. axillaris accessorius* in 30 min. With prolonged time, the fluorescent signal became stronger. In addition, fluorescent signals were detected in other organs of the mice 60 min later. The results demonstrate that the NLG919/IR780 micelles can not only migrate to the lymphatic system and accumulate in the lymphatic nodes but can also enter into the body circulation (Figure 9C,D). Similar phenomena and results were found following intratumoral injection of NLG919/IR780 micelles. After being injected intratumorally, the FI of the nearest lymphatic node, *Ln. subiliacus* in this case, reached the maximum value 60 min later. Meanwhile, fluorescent signals were detected in other organs as well (Figure 9E,F).

**Figure 10:**
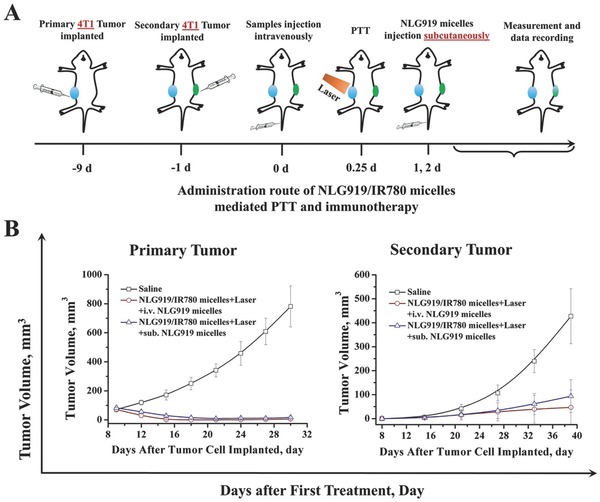
Effect of subcutaneous administration of NLG919 micelles after PTT on the tumor growth of primary and secondary tumors. A) Administration route. B) Tumor volume variation of primary and secondary tumors, respectively.

*Suppression of Primary Tumor and Secondary Distal Tumor In Vivo after Subcutaneous Administration*: The IDO is highly expressed not only in the tumor but also in APCs. Therefore, we designed the NLG919/IR780 micelles to circulate in the lymphatic system for IDO inhibition by NLG919 and for targeting to the tumor via EPR for efficient PTT. From the results of fluorescent imaging assays in vivo, we can conclude that the NLG919/IR780 micelles can fulfill this demand. To prove this, we established a 4T1 breast cancer model in balb/c mice. After the grown tumors have been treated with NLG919/IR780‐micelle‐mediated PTT, parts of the tumor‐bearing mice were administered subcutaneously with NLG919 micelles each day for the next two days, while the remaining mice were still intravenously administered NLG919 micelles (**Figure** **10**A). The results reveal that no significant difference in the tumor growth was found between the subcutaneously administered group and the intravenously administered group (Figure 10B).

## 3 Conclusion

In summary, we first evaluated the effect of mild heating on the growth rate of tumor cells in vitro and in vivo. We observed that the tumor margin beyond effective PTT proliferated faster than the untreated tumor due to the upregulation of HSP, IDO, PD‐L1, etc. To efficiently inhibit the growth of the localized tumor and stimulate the host to generate an immune response to suppress the growth of the tumor margin beyond effective PTT and the distal tumor, we have developed a nanomedicine containing NIR photosensitizer (IR780) and IDO inhibitor (NLG919) to realize tumor photothermal therapy and immunotherapy. Through in vitro cellular assay, we confirmed that the NLG919/IR780 micelles can inhibit the activity of IDO but do not suppress the IDO expression. When the treatment was combined with PTT, the tumor cell growth was efficiently inhibited in vitro and in vivo. We also proved that the NLG919/IR780 micelles can accumulate in the tumor site via passive targeting and migrate to the lymphatic system to enter the lymphatic circulation. By investigating the antitumor performance in vivo, we found that the NLG919/IR780‐micelle‐mediated PTT and immunotherapy efficiently inhibited the primary tumor, suppressed the growth of secondary tumor, and increased the infiltrated T cells in tumor tissue, favoring the differentiation of T cells to CD8^+^ T cells. Combined with the suppression of Treg activity, NLG919/IR780‐micelle‐mediated PTT and immunotherapy showed a therapeutic outcome. The results demonstrated that the NLG/IR780 micelles enable the suppression of the tumor margin beyond effective PTT and strengthen the immune response to inhibit the distal tumor.

## 4 Experimental Section

*Material*: Methoxy poly (ethylene glycol) (MPEG‐OH, Mn = 2000), stannous octoate (Sn(Oct)2), *∂*‐lipoic acid, tetradecanol, pentadecanol, hexadecanol, and *ɛ*‐caprolatone were purchased from Sigma‐Aldrich (Saint Louis, USA). Photosensitizer IR780 was purchased from Sigma‐Aldrich. IDO inhibitor NLG919 was purchased from Selleck. CD3‐FITC monoclonal antibody (catalog: 11‐0032‐80, clone: 17A2), CD4‐APC monoclonal antibody (catalog: 17‐0041‐82, clone: GK1.5), and CD8a‐PE‐cyanine7 monoclonal antibody (catalog: 25‐0081‐82, clone: 53–6.7) were purchased from Invitrogen (eBioscience, Invitrogen, Thermo Fisher Scientific, Massachusetts, USA). IDO monoclonal antibody (rabbit anti‐IDO, catalog: bs‐15493R), HSP 70 monoclonal antibody, and HSP 90 monoclonal antibody (Monoclonal mouse IgG2B clone# 341320, catalog: MAB3286, R&D) were purchased from Bioss Antibodies and R&D, respectively. 3‐(4,5‐dimethyl‐2‐thiazolyl)‐2,5‐diphenyl‐2H‐tetrazolium bromide (methyl thiazolyltetrazolium) (MTT) were obtained from Sigma‐Aldrich (Saint Louis, USA). The T_reg_ Flow Kit (True‐Nuclear one step staining mouse T_reg_ Flow kit (FOXP3 Alexa FluorR 488/CD25 PE/CD4 PerCP)) was obtained from Biolegend. Dulbecco's modified Eagle's medium (DMEM), penicillin‐streptomycin liquid (100X), and fetal bovine serum (FBS) were purchased from HyClone (Logan, USA). Ethanol was purchased from Aladdin (Shanghai, China).

293T, A549, LL2, MCF‐7, and 4T1 cells were purchased from American Type Culture Collection (Rockville, MD) and were cultured with DMEM supplement with 10% FBS, 100 U mL^−1^ penicillin, and 100 µg mL^−1^ streptomycin, respectively. The cell cultures were maintained in an incubator at 37 °C with a humidified 5% CO_2_ atmosphere.

Female BALB/c‐nu mice (20 ± 2 g) and BALB/c mice (16 ± 2 g) were purchased from HFK Bioscience Co., Ltd. (Beijing, China) and kept under specific pathogen free (SPF) condition with free access to standard food and water. All animal procedures were performed following the protocols approved by the Institutional Animal Care and Treatment Committee of Sichuan University (Chengdu, P. R. China).

*Preparation of NLG919/IR780 Micelles*: The micelles were all prepared by the thin‐film hydration method described elsewhere.^43^ In general, first, NLG919 (6 mg) (or plus 2 mg of IR780) and MPEG‐PCL (42 mg) were codissolved in ethanol in a solanum bottle. Next, the ethanol was evaporated at 62 °C under reduced pressure. After 15 min, once the ethanol was totally evaporated, preheated water (5 mL) was added to rehydrate the polymer‐drug matrix. A clear micellar dispersion was obtained. The obtained dispersion of micelles was filtered through a 0.22‐µm membrane filter for further applications.

*Characterization of DTX/IR780 Micelles*: Drug loading and encapsulation efficiency of IR780 and NLG919‐mPEG‐PCL micelles were quantified by UV–vis spectrometer, respectively.

The particle size and zeta potential of drug‐loaded micelles were determined by dynamic light scattering (Nano‐ZS 90, Malvern Instruments, Malvern, UK) at a constant temperature of 25 °C. Each experiment was performed in triplicate, and the data were indicated as the mean ± standard deviation (SD).

The absorbance spectrum of the Free IR780 or IR780 micelles was measured by UV–vis photosentimeter (PE, USA).

Release of NLG919 and IR780 from the micelles was determined by dialysis using a membrane with a molecular weight cut‐off of 8–14 KDa. A total of 1 mL of micelle dispersion (1 mg mL^−1^) was placed into a dialysis bag and dialyzed against 30 mL of PBS (pH = 7.4, 0.2 m) containing 0.5% (w/w) of Tween‐80 at 37 °C with gentle shaking. At predetermined time points, the incubation medium was collected and replaced with fresh medium. The amount of NLG919 or IR780 released was quantified by measuring the NLG919 or IR780 concentration in the collected medium via UV–vis spectrophotometer in scan mode (scan range: 300–900 nm, SHIMADZU, UV‐2600, the samples were mixed with ethanol before measurement). The experiment was performed in triplicate, and the data were indicated as the mean ± SD.

*Photothermal Performance of NLG919/IR780 Micelles*: The photothermal conversion of IR780 or IR780 micelles was evaluated by detecting the temperature variation of the IR780 solution or IR780 micelles dispersion (1 mL) under the irradiation with 808 nm laser. The concentrations of IR780 were 0, 25, 50, and 100 µg mL^−1^. The laser power was settled at 1.0 W cm^−2^, and the temperature was detected by a digital thermometer and a Fluke infrared thermal imaging system (Ti‐32, Fluke).

*IDO Activity Inhibition by NLG919/IR780 Micelles In Vitro*: IDO activity inhibition by NLG919/IR780 micelles in vitro was investigated by measuring the survival of the 293T cell lines and MCF‐7 cell lines with or without IDO transfection after being cocultured with NLG919/IR780 micelles. In brief, 293T or MCF‐7 cells were seeded in a 96‐well plate with 5 × 103 cells per well. After being cultured for 24 h, the cells were coincubated with different concentrations of NLG919/IR780 micelles. A total of 24 h later, the medium was replaced by 100 µL of 0.5 mg mL^−1^ MTT, and 3 h later, it was replaced by 100 µL of dimethyl sulfoxide (DMSO). The absorbance was measured at 570 nm using an infinite M200 microplate reader (Tecan, Durham, USA). Untreated cells in the medium were used as a control. All experiments were carried out in quadruplicate. In parallel, in the case of IDO transfection, after the cells were seeded in a 96‐well plate and cultured for 24 h, the IDO gene was transfected into the cells by lipofectamine 3000. A total of 4 h later, the culturing medium was replaced by fresh medium, and 12 h later, the cells were cocultured with NLG919/IR780 micelles. The cell survival was measured as described above.

*Anticancer Evaluation of NLG919/IR780 Micelles In Vitro*: MCF‐7 cells were seeded at 5 × 103 cells per well in a 96‐well plate, preincubated for 24 h, then incubated with NLG919/IR780 micelles for 24 h at NLG919 concentrations ranging from 0 to 100 µg mL^−1^. Next, the medium was replaced by 100 µL of 0.5 mg mL^−1^ MTT, and the MTT solution was replaced by 100 µL of DMSO solution after 3 h. The absorption was measured at 570 nm with a reference wavelength of 630 nm using an infinite M200 microplate reader (Tecan, Durham, USA). Untreated cells in the medium were used as a control. All experiments were carried out in quadruplicate.

Laser irradiation was introduced to evaluate the in vitro anticancer performance of PTT/IDO inhibition combination therapy of breast cancer cells. In this assay, the cells that had been preincubated for 24 h were then incubated with NLG919/IR780 micelles. Four hours later, the culture medium of each sample was replaced with fresh DMEM culture medium with penicillin‐streptomycin after rinsing once with PBS buffer. Next, the 808 nm laser was used for irradiation at a power of 1.0 W cm^−2^ and an irradiation time of 5 min.

In the case of IDO transfection, the procedure was performed using the procedure for IDO transfection and laser irradiation as described above.

*Western Blotting Assays for Protein Expression Characterization*: The expression levels of IDO, HSP 70, HSP 90, and PD‐L1 were identified by Western blotting assays. Western blot analyses were performed according to the protocols for the routine with antibodies against IDO, HSP 70, HSP 90, PD‐L1, and β‐actin, respectively.

*Fluorescence Imaging of MCF‐7 Tumors In Vivo*: The enrichment of NLG919/IR780 micelles in MCF‐7 tumors was indirectly evaluated by fluorescence imaging. IR780 was used as an NIR fluorescent dye. The MCF‐7 tumor model was established by subcutaneously inoculating 1 × 107 MCF‐7 cells into the right flank of each nude mouse. When the tumor volume reached 120 mm^3^, free IR780 and NLG919/IR780 micelles (1 mg kg^−1^ b.w. of IR780) were intravenously injected into the mice. Near‐infrared imaging was carried out at predetermined time points using a Maestro in vivo spectrum imaging system (CRI, Woburn, MA, USA; excitation = 740 nm, emission = 790 nm long pass).

*Migration of NLG919/IR780 Micelles to the Lymphatic System*: To evaluate the migration of NLG919/IR780 micelles to the lymphatic system, three models were established. The balb/c mice were divided into four groups. The NLG919/IR780 micelles were injected into the fat pads of the feet, hands and tumors (4T1 breast cancer model) of the mice, respectively. One group of mice was injected with free IR780/NLG919 into the fat pads of the feet and was used to compare with the micelle‐treated group. At different time points, the fluorescent images and intensity were collected by IVIS fluorescent imaging system.

*Pharmacokinetic Evaluation of NLG919/IR780 Micelles*: Ten female Balb/c mice at 6–8 weeks old were divided into two groups and administered intravenously with free NLG919 (dissolved in ethanol then dispersed in tween‐80 aqueous solution) and NLG919/IR780 micelles at a dose of 25 mg NLG919 per kg body weight. Blood samples of 100 µL were withdrawn from the retro‐orbital plexus/sinus of the mice at predetermined timepoints (0.5, 1, 2, 4, 8, 12, 24, and 48 h) in heparinized tubes, and the blood samples were centrifuged at 800 g for 15 min. The NLG919 in the serum samples was extracted twice by dichloromethane (2 mL each time) and dried under airflow. Then, the samples were redissolved by methanol, and the mixtures were centrifuged at 10 000 g for 8 min. The supernatants were collected from each sample and the NLG919 contents were determined by an LC‐MS system (PerkinElmer LX‐50/Qsight).

*In Vivo Photothermal Performance Investigation*: 4T1 breast cancer‐bearing balb/c mice were used to evaluate the photothermal performance of NLG919/IR780 micelles in vivo. After the intravenous administration of saline and NLG919/IR780 micelles, the mice were anesthetized and then irradiated by an 808 nm laser with a power of 1.0 W cm^−2^ for 5 min. The temperature of the tumor site was recorded by an infrared imaging device (Fluke, T32, USA).

*Evaluation of In Vivo Tumor Growth Inhibition*: Two kinds of breast cancer models were established in vivo for different evaluations. For example, to investigate the NLG919/IR780‐micelle‐mediated tumor PTT and immunotherapy in vivo, a 4T1 (or 4T1‐LUC) tumor model was established in Balb/c mice. When the mean volume of primary tumor, established by direct subcutaneous injection of 4T1 cells (1 × 10^6^ per mouse) into the right flank of the mice, reached approximately 100 mm^3^, 1 × 10^6^ 4T1 cells per mouse were subcutaneously injected into the right flank of the mice. The mice were randomly double‐blindly divided into 5 groups (*n* = 6) and intravenously administered with NS, NLG919 micelles, IR780 micelles + Laser, NLG919/IR780 micelles, and NLG919/IR780 micelles + Laser (NLG919: 6 mg kg^−1^ body weight, IR780: 2 mg kg^−1^ body weight). Laser power was settled at 1.0 W cm^−2^, and the irradiation time was 5 min. The tumor volumes of both flanks and body weights were measured every other day.

The MCF‐7 breast cancer model was also established in balb/c‐nu mice. The administration route was similar to that used in the 4T1 cell model.

*The Mechanism Study of Immunotherapy*: To study the immune cells, particularly the CD4^+^ and CD8^+^ T cells, in secondary tumors, tumors were eviscerated from mice in different groups and stained with anti‐CD3‐FITC (Invitrogen, catalog: 11‐0032‐80, clone: 17A2), anti‐CD8a‐PE‐cyanine (Invitrogen, catalog: 25‐0081‐82, clone: 53–6.7) and anti‐CD4‐APC (Invitrogen, catalog: 17‐0041‐82, clone: GK1.5) antibodies according to the manufacture's protocols. Briefly, the eviscerated tumor tissues were cut into small pieces (1 × 1 mm^2^) and kept in a glass homogenizer containing PBS with 2% of heat‐inactivated fetal bovine serum. By gently pressing the homogenizer without addition of digestive enzyme, a single‐cell suspension was obtained. The obtained cells were stained with specific fluorescence‐labeled antibodies. CD8^+^ T lymphocytes and CD4^+^ helper T cells were CD3^+^CD4^+^CD8^+^ and CD3^+^CD4^+^CD8^−^, respectively. The populations of T_reg_ in the spleen tissues were identified by flowcytometry using a T_reg_ Flow kit. The antibody concentrations were settled according to the manufacturer's protocols.

*Immunohistochemistry (IHC)*: Tumor tissues were immunostained with mouse antibodies for IDO or PD‐L1. Secondary goat antimouse immunoglobulin G (IgG) was applied after washing with tris‐buffered saline. Reactions were developed with 3,3′‐diaminobenzidine chromogen. Appropriate negative controls for the immunostaining were also prepared by omitting the primary antibody step and substituting it with nonimmune mouse serum.

*Statistics*: Statistical analysis was performed using the SPSS 15.0 software (IBM Corporation, Armonk, NY, USA). The results were indicated as the mean ± SD. Analysis of variance (ANOVA) was employed for multiple group comparisons, and results of *p* < 0.05 were considered statistically significant.

## Conflict of Interest

The authors declare no conflict of interest.

## Supporting information

Supplementary
