# Supplementary material for: Photosensitizer Micelles Together with IDO Inhibitor Enhance Cancer Photothermal Therapy and Immunotherapy
Source: Adv Sci (Weinh). 2018 Feb 26;5(5):1700891. doi: 10.1002/advs.201700891 (PMC5979747; doi:10.1002/advs.201700891)
Supplement: Supplementary file 1 — Supplementary [file ADVS-5-1700891-s001.pdf]

Copyright WILEY-VCH Verlag GmbH & Co. KGaA, 69469 Weinheim, Germany, 2018.

ADVANCED SCIENCE
Open Access

Supporting Information

for *Adv. Sci.,* DOI: 10.1002/advs.201700891

Photosensitizer Micelles Together with IDO Inhibitor Enhance Cancer Photothermal Therapy and Immunotherapy

*Jinrong Peng, Yao Xiao, Wenting Li, Qian Yang, Liwei Tan, Yanpeng Jia, Ying Qu, and Zhiyong Qian**

Supporting Information

Photosensitizer micelles together with IDO inhibitor enhance cancer photothermal therapy and immunotherapy

*Jinrong Peng, Yao Xiao, Wenting Li, Qian Yang, Liwei Tan, Yanpeng Jia, Ying Qu, Zhiyong Qian**

Dr. Jinrong Peng, Yao Xiao, Yanpeng Jia, Dr. Ying Qu, Prof. Zhiyong Qian
Stake Key Laboratory of Biotherapy/Collaborative Innovation Center of Biotherapy, West China Hospital, Sichuan University, Chengdu, Sichuan, PR China
E-mail: anderson-qian@163.com
Dr. Qian Yang
School of Pharmacy, Chengdu Medical College, No. 783, Xindu Avenue, Xindu District, Chengdu, 610500, Sichuan, P. R. China
Dr. Wenting Li and Dr. Liwei Tan
Department of Pharmacy, West China Second University Hospital, No. 20, People's Southern Road, Chengdu, 610041

Keywords: nanoparticle, ineffective photothermal therapy, IDO inhibitor, immunotherapy, combinational therapy

Figure S1

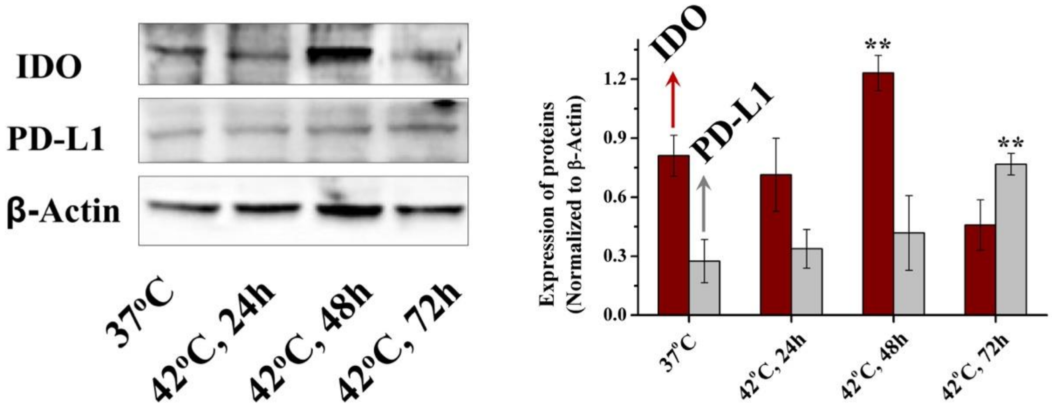

Figure S1 the duration time of up-regulated proteins (IDO, PD-L1) after heat treatment.

Figure S2

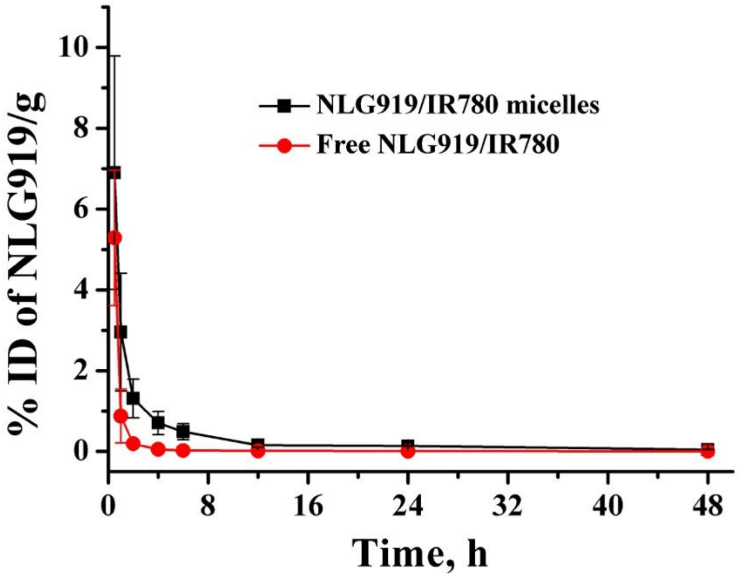

Figure S2 the pharmacokinetic profiles of NLG919/IR780 micelles and free form NLG919/IR780, respectively.

Raw data of western blotting

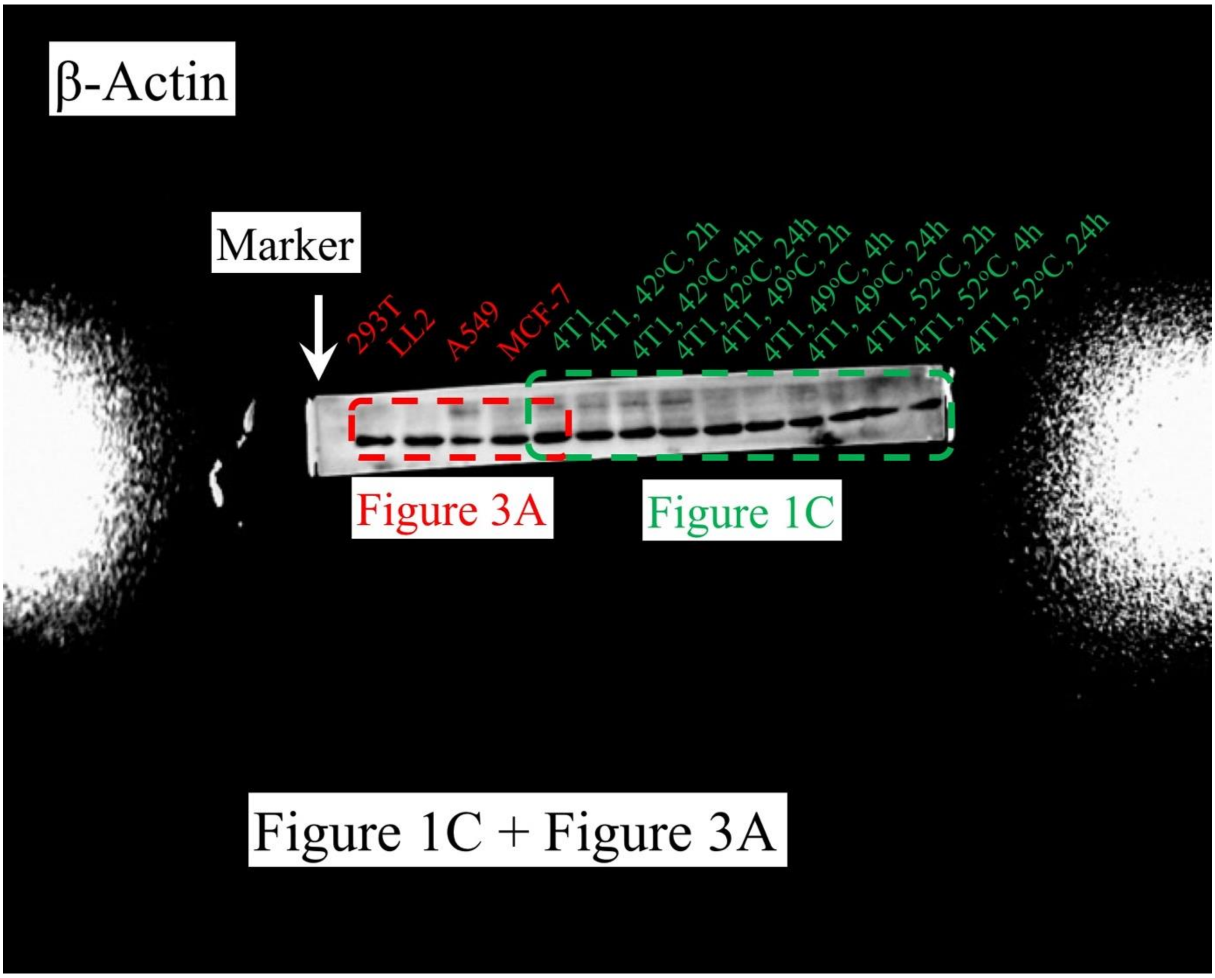

Supplementary Figure 1. The raw data of Figure 1C and Figure 3A, evaluated by western blotting. In here, the image of actin was listed and marked. The other images were uploaded as separated images. In order to obtain desired imaging quality, we have cutted the gels of the target proteins from the whole gels for imaging. The expression levels of each protein were normalized to β-actin, and listed in figure 3B, and Figure 1D and 1E, respectively.

Raw data of western blotting

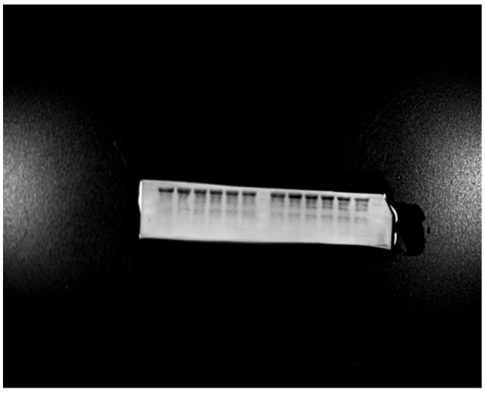

IDO

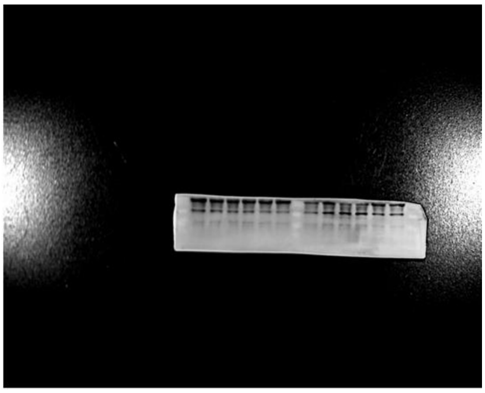

β-Actin

Supplementary Figure 2. The expressions of IDO, Actin of 4T1 cells, after co-culturing with different concentrations of NLG919 micelles, respectively, evaluated by western blotting. (Figure 3E)

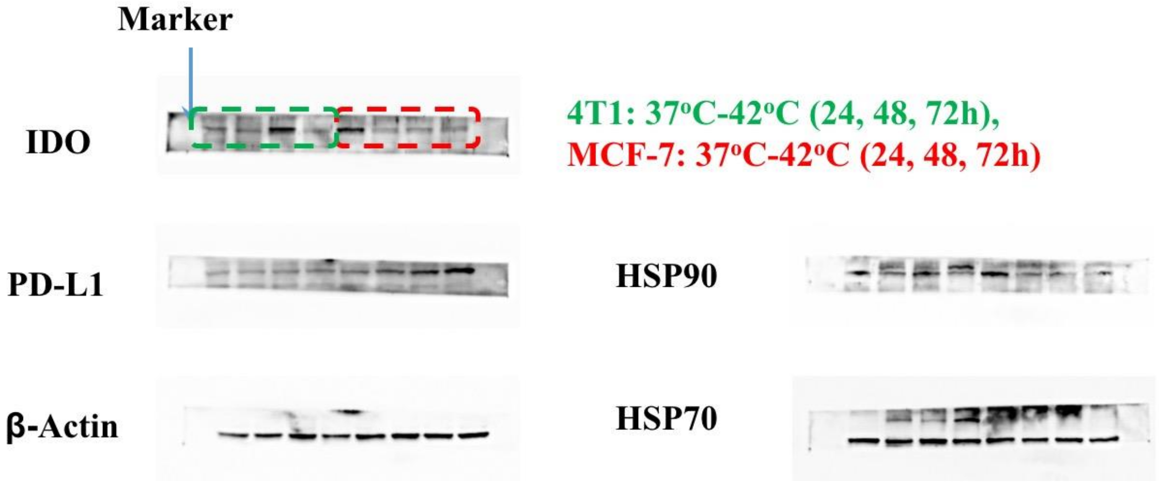

Supplementary Figure 3. The raw data of Figure S 1. the duration time of up-regulated proteins (IDO, PD-L1) after heat treatment.
